# Highly Thermally Conductive Liquid Crystalline Epoxy Resin Vitrimers with Reconfigurable, Shape‐Memory, Photo‐Thermal, and Closed‐Loop Recycling Performance

**DOI:** 10.1002/advs.202410362

**Published:** 2024-11-22

**Authors:** Fengyuan Zhang, Junliang Zhang, Kuan Zhang, Xiao Zhong, Mukun He, Hua Qiu, Junwei Gu

**Affiliations:** ^1^ Shaanxi Key Laboratory of Macromolecular Science and Technology School of Chemistry and Chemical Engineering Northwestern Polytechnical University Xi'an Shaanxi 710072 P. R. China

**Keywords:** closed‐loop recycling, epoxy resin vitrimers, liquid crystalline epoxy, photo‐thermal effect, thermal conductivity

## Abstract

The low thermal conductivity, poor toughness, and non‐reprocessability of thermosetting epoxy resins severely restrict their applications and sustainable development in flexible electronics. Herein, liquid crystalline epoxy (LCE) and dynamic ester and disulfide bonds are introduced into the cured network of bisphenol A epoxy resin (E‐51) to construct highly thermally conductive flexible liquid crystalline epoxy resin (LCER) vitrimers. LCER vitrimers demonstrate adjustable mechanical properties by varying the ratio of LCE to E‐51, allowing it to transition from soft to strong. Typically, a 75 mol% LCE to 25 mol% E‐51 ratio results in an in‐plane thermal conductivity (*λ*) of 1.27 W m^−1^ K^−1^, over double that of pure E‐51 vitrimer (0.61 W m^−1^ K^−1^). The tensile strength and toughness increase 2.88 folds to 14.1 MPa and 2.45 folds to 20.1 MJ m^−3^, respectively. Besides, liquid crystalline phase transition and dynamic covalent bonds enable triple shape memory and three‐dimensional shape reconstruction. After four reprocessing cycles, *λ* and tensile strength remain at 94% and 72%, respectively. Integrating carbon nanotubes (CNTs) imparts photo‐thermal effect and enables “on” and “off” switch under near‐infrared light to LCER vitrimer. Furthermore, the CNTs/LCER vitrimer displays light‐induced actuation, self‐repairing, and self‐welding besides the closed‐loop recycling and rapid degradation performance.

## Graphical abstract

The combination of liquid crystalline epoxy and dynamic covalent bonds creates highly thermally conductive liquid crystalline epoxy resin (LCER) vitrimers with multi‐shape memory and closed‐loop recycling capabilities. The 0.5 wt% introduced carbon nanotubes enable LCER vitrimers with photothermal conversion and near‐infrared light‐induced actuation, providing insights for designing recyclable materials with special functions, such as soft actuators and intelligent wearable electronics.

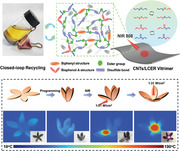

## 1 Introduction

Due to the accelerating advancements in the fifth generation (5G) mobile communication and Internet technologies, flexible electronics are attracting tremendous interest in the fields of wearable devices and soft robotics.^[^ ^1^, ^2^, ^3^ ^]^ However, the increasing integration and power density make it a considerable challenge for heat dissipation.^[^ ^4^, ^5^, ^6^ ^]^ Flexible polymer materials with high thermal conductivity are ideal for efficiently dissipating the accumulated heat to maintain the normal operation of electronic devices.^[^ ^7^ ^]^ Epoxy resins are considered to be excellent substrate and encapsulation materials for electronic devices due to the great advantages of low curing shrinkage, good mechanical strength, insulating properties, and chemical resistance.^[^ ^8^, ^9^ ^]^ However, conventional epoxy resins form a disordered cross‐linking network after curing which leads to intense phonon scattering. Therefore, their intrinsic thermal conductivity (*λ*) is usually as low as ≈0.2 W m^−1^ K^−1^, while their high rigidity and low elongation make them incapable of utilization in flexible electronic devices.^[^ ^10^ ^]^

Since intrinsic *λ* is related to the multilevel structure of polymers, constructing a regular and ordered structure capable of increasing the phonon mean free path (MFP) is vital to improve the intrinsic *λ* of polymers.^[^ ^11^ ^]^ Building phonon conduction paths through the introduction of liquid crystalline units by forming ordered orientation in the cross‐linked network is currently recognized as an effective means.^[^ ^12^, ^13^ ^]^ The liquid crystalline units can be self‐assembled into oriented domains by π–π stacking in the molten state or in solution.^[^ ^14^ ^]^ When these crystalline domains become larger in size and interconnect to form phonon conduction pathways, the MFP increases and leads to enhanced thermal conductivity. For instance, Giang et al.^[^ ^15^ ^]^ synthesized a methylenediamine liquid crystalline epoxy monomer, which was cured by 4,4′‐diaminodiphenylsulfone. The obtained liquid crystalline epoxy resins showed a *λ* of 0.45 W m^−1^ K^−1^. In our previous work, Gu et al.^[^ ^16^ ^]^ designed and synthesized a main‐chain liquid crystalline epoxy monomer with biphenyl mesogens. The epoxy resin cured with 4,4′‐diaminodiphenylmethane displayed a notable *λ* of 0.51 W m^−1^ K^−1^.

The introduction of liquid crystalline units can not only significantly improve the thermal conductivity of the epoxy resin, but also endow it with excellent mechanical properties and unique phase transition functions. Therefore, liquid crystalline epoxy resins are also applied to prepare flexible actuators with shape memory.^[^ ^17^ ^]^ The key to realize the shape memory process lies in the arrangement of liquid crystalline units, and this orderly arrangement of liquid crystalline units is usually accomplished by chemical cross‐linking. However, the shape of the material and the driving process are permanently fixed and cannot be changed once the arrangement process is completed. In addition, due to the thermosetting characteristic, traditional epoxy can only be filled or replaced once damaged to maintain the working condition of the device. Furthermore, the waste epoxy cannot be recycled, which not only causes a waste of resources, but also causes burdens to environment.^[^ ^18^, ^19^ ^]^ Hence, design and preparation of highly thermally conductive epoxy resin with reprocessable and recyclable performance are of significant value in advancing the 5G electronics industry and sustainable development.

Introducing dynamic covalent bonds into the cured network to prepare liquid crystalline epoxy resin vitrimer can effectively solve the aforementioned problems.^[^ ^20^, ^21^ ^]^ Epoxy resin vitrimer is a class of materials combining the unique properties of thermoset and thermoplastic. Below the dynamic covalent bonds exchange temperature (*T*_v_), the material demonstrates a stable traditional thermoset cross‐linked network with excellent mechanical, chemical, and friction properties. Whereas, the dynamic covalent bonds can be topologically reconfigured through exchange reaction at temperature above the *T*_v_, thus realizing the functions of reprocessing, shape‐morphing, self‐repair, and degradation.^[^ ^22^, ^23^ ^]^ Since the forming and breaking of covalent bonds progress simultaneously, it ensures that the cross‐linking density and the number of covalent bonds during exchange are in dynamic equilibrium, so as to realize recycling.^[^ ^24^, ^25^ ^]^ For instance, Zhou et al.^[^ ^26^ ^]^ prepared a dual reversible cross‐linking network, which showed excellent recycling and reprocessing properties due to the synergistic effect of disulfide bond and dynamic ester bond. Chen et al.^[^ ^27^ ^]^ prepared a difunctional epoxide‐based polyurethane containing dynamically hindered urea bonds, which showed elastic shape memory, three‐dimensional (3D) permanent shape reconstruction capability, and excellent recyclability. However, activation of dynamic covalent bond exchange usually requires a certain temperature and pressure. It is always difficult to realize directly and local control using temperature and pressure in some high‐tech precision devices.^[^ ^28^ ^]^ Therefore, it is essential to activate the dynamic covalent bond exchange through some other stimulus responses that are easy to operate, precisely controlled, and less damaging caused to the device.^[^ ^29^ ^]^ Among various stimuli, light is a powerful and versatile physical stimulus due to its cost‐effectiveness, wireless actuation, and fast response.^[^ ^30^, ^31^ ^]^ Introducing photo‐thermal fillers into polymer matrices to prepare photo‐thermally responsive materials enables remote, localized, and non‐contact control, triggering the response “on demand” and thus reducing the need to intervene in the surrounding environment.^[^ ^32^, ^33^ ^]^ This simple and effective stimulus‐response is important for 3D printing of complex geometries, non‐removable parts, and localized self‐healing of precision instruments.^[^ ^34^ ^]^

**Figure 1:**
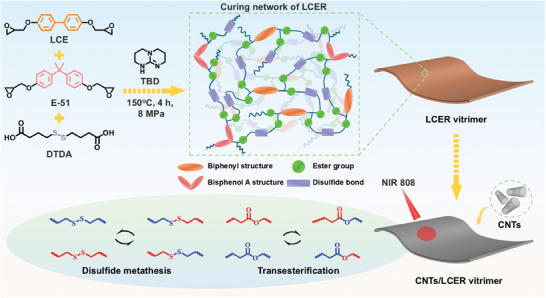
Preparation of LCER and CNTs/LCER vitrimers and the mechanism of network rearrangement enabled by disulfide metathesis and carboxylate transesterification.

Currently, it is still very challenging to make epoxy resins simultaneously with high inherent thermal conductivity and excellent reprocessing and recycling performance, which has rarely been reported so far. In this work, highly thermally conductive liquid crystalline epoxy resin (LCER) vitrimers are prepared by introducing biphenyl mesogens and dual dynamic disulfide and ester bonds into the cured network (**Figure** **1**). First, a liquid crystalline epoxy (LCE, Figure S1, Supporting Information) monomer was synthesized through a convenient one‐step reaction of 4,4′‐dihydroxybiphenyl and epichlorohydrin (Scheme S1, Supporting Information). Then, 4,4′‐dithiobisdibutyric acid (DTDA) was employed to cure LCE and bisphenol A epoxy (E‐51) to construct the covalent adaptable liquid crystalline cross‐linked network. The effects of the ratio of LCE and E‐51 (Table S1, Supporting Information) on the intrinsic thermal conductivity and mechanical properties of LCER vitrimers were investigated. LCER vitrimers showed excellent reprocessing and shape memory properties. Besides, LCER vitrimers could realize programming, self‐repairing, and self‐welding under near‐infrared (NIR) light radiation due to the photothermal effect of the introduced 0.5 wt% carbon nanotubes (CNTs) while being able to be closed‐loop recycled and rapidly degraded. This study not only broadens the applications of epoxy resins, providing insights for designing and fabrication of multifunctional epoxy resin vitrimers with high intrinsic thermal conductivity utilized in flexible wearable electronics and soft actuators, but also contributes to reducing environmental pollution and advancing sustainability.

## 2 Results and Discussion

### 2.1 Cross‐Linking Network of LCER Vitrimers

**Figure 2:**
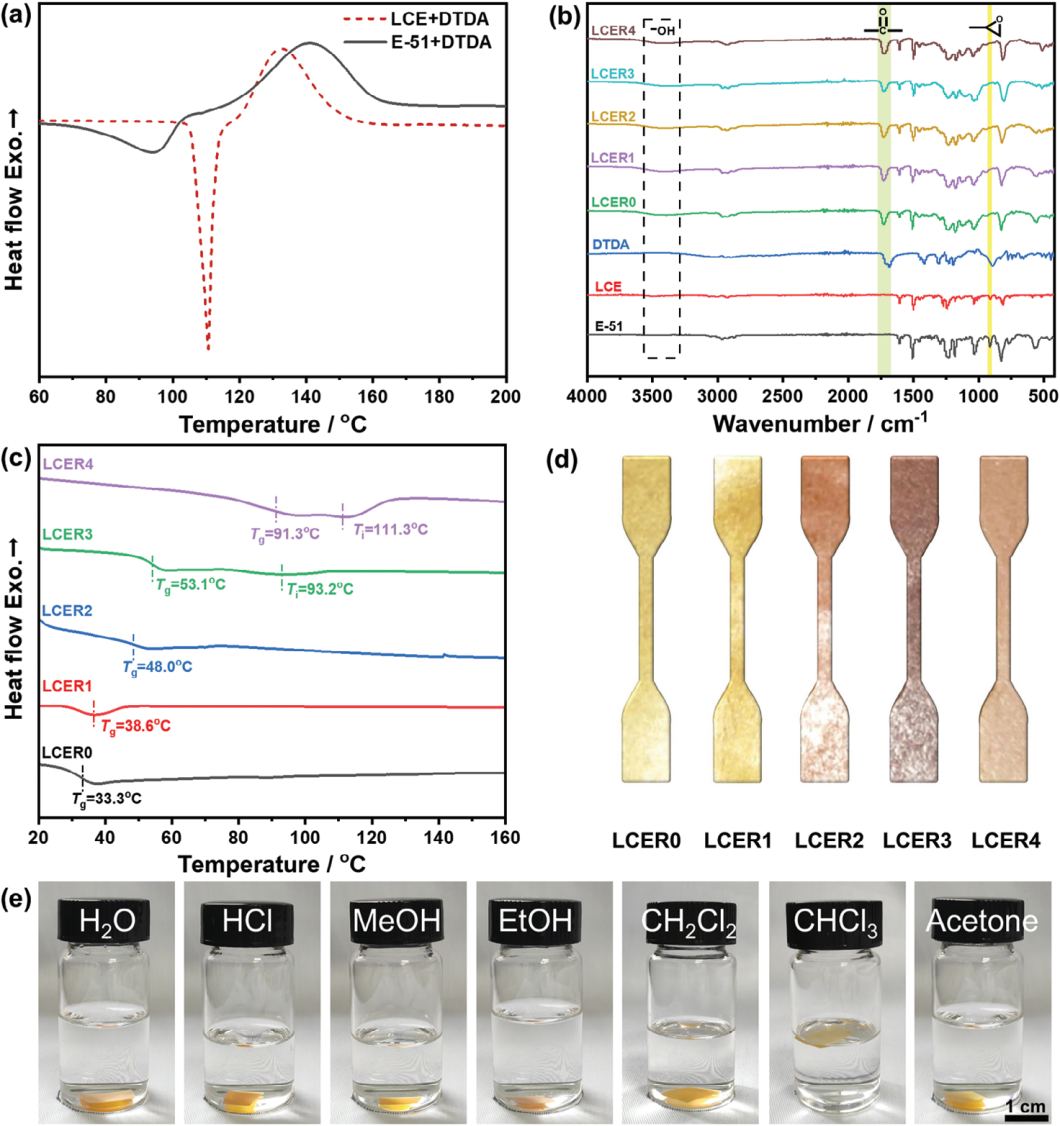
DSC curves of the curing reaction of LCE or E‐51 with DTDA (a); FT‐IR spectra of E‐51, LCE, DTDA, and LCER0‐4 (b); DSC curves of LCER0‐4 (c); tensile specimens of LCER0‐4 (d); digital photos of LCER3 after immersion in different solvents for 2 weeks at room temperature (e).

The curing temperature has a crucial impact on the properties of the liquid crystalline epoxy resin, for instance, the mechanical properties and thermal conductivity. In order to maximally retain the ordered structure within the cross‐linking network so as to minimize the phonon scattering, the curing temperature should be within the temperature window for the formation of liquid crystalline.^[^ ^35^, ^36^ ^]^ As shown in **Figure** **2**a, both LCE and E‐51 with DTDA exhibited an exothermic peak between 120 and 160 °C, indicating that the curing reaction in this interval was the intensest. Considering the liquid crystalline formation interval was between 147 and 155 °C as shown in the differential scanning calorimetry (DSC) curves and polarizing optical microscope (POM) images of LCE (Figure S2, Supporting Information), 150 °C was selected as the curing temperature for LCER vitrimers.

The cross‐linking network of LCER vitrimers was first analyzed by Fourier transform infrared (FT‐IR) spectroscopy. As displayed in Figure 2b, the peak at 913 cm^−1^ attributed to epoxy group disappeared completely whereas the signal at 3430 cm^−1^ corresponding to the hydroxyl group generated from ring‐opening of epoxide appeared instead. Besides, the peak at 1700 cm^−1^ ascribed to carboxyl group of DTDA was not visible anymore, which was replaced with the generated stretching vibration peak of ester bond at 1720 cm^−1^. Furthermore, no melting peak or exothermic peak was present in the DSC curves of the LCER vitrimers as depicted in Figure 2c. FT‐IR and DSC analyses indicated the complete curing of the vitrimers. Additionally, the glass transition temperature (*T*_g_) of LCER vitrimers increased significantly with the increase of LCE content from 33.3 °C for LCER0 to 91.3 °C for LCER4. It is noteworthy that LCER3 and LCER4 also showed an isotropization temperature (*T*_i_) at 93.2 and 111.3 °C, respectively. This is, on one hand, due to the increased rigidity of the cross‐linked network caused by the introduction of biphenyl mesogens, thus increasing the temperature at which the chain segments started moving. On the other hand, the orderness of the cross‐linked network was enhanced as LCE increased, resulting in the anisotropic to isotropic transition. The vitrimers also displayed a decrease transparency from LCER0 to LCER4 as observed from their digital images (Figure 2d), indicating an enhanced scattering of visible light due to the increased crystallinity.^[^ ^37^ ^]^

The degree of cross‐linking of LCER vitrimers was further analyzed through the investigation of gel content and swelling ratio. As shown in Table S2 (Supporting Information), LCER vitrimers revealed a high gel content (>96%) and a low swelling ratio (<0.7%), suggesting that the networks were fully cross‐linked. The resistance to different solvents, such as water, hydrochloric acid, methanol, ethanol, dichloromethane (DCM), chloroform, and acetone was exemplified by LCER3 (Figure 2e). After soaking in the solvent for two weeks at room temperature, all the samples remained intact, indicating that LCER vitrimers displayed good resistance to organic and acidic solvents.

### 2.2 Thermal Conductivities of LCER Vitrimers

**Figure 3:**
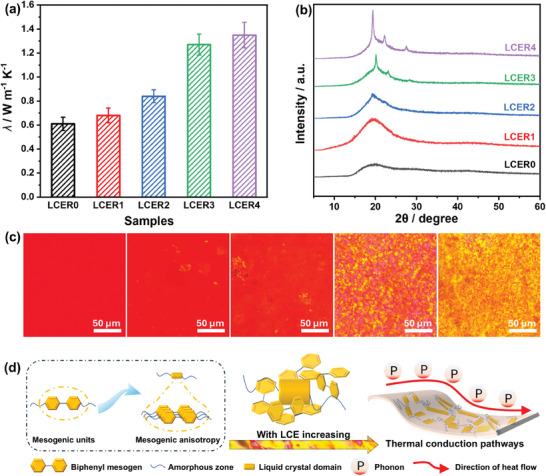
The *λ* values (a), XRD curves (b), and POM images at room temperature (c) of LCER0‐4; schematic of thermal conduction mechanism within LCER vitrimer (d).

As depicted in **Figure** **3**a, the in‐plane thermal conductivity (*λ*) exhibited a significant increase from 0.61 W m^−1^ K^−1^ for LCER0 to 1.27 W m^−1^ K^−1^ for LCER3 and 1.35 W m^−1^ K^−1^ for LCER4. The enhancement of thermal conductivity with the addition of LCE is primarily attributed to the increased ordering of the cured network. This is because the biphenyl mesogenic units would self‐assemble into highly ordered structures driven by the strong π–π stacking to generate interconnected crystal‐like domains within the curing network and form continuous phono transmission paths. Moreover, the π–π stacking of biphenyl mesogens increased the rigidity of molecular chains, reducing ineffective molecular vibrations or folding. The above effects suppressed phonon scattering and segmental rotation but increased the MFP of the phonons and phonon group velocity, allowing heat to be more effectively transferred.^[^ ^38^ ^]^ However, when the content of LCE was less than 50 mol%, the locally ordered crystal domains were surrounded by the amorphous regions formed by E‐51, creating the “sea‐island” structure, which was not conducive to phonon transmission. This can explain the minor enhancement of thermal conductivity from LCER0 to LCER1 (0.68 W m^−1^ K^−1^).

The microstructures of LCER vitrimers were further investigated by X‐ray diffraction (XRD) and POM to confirm the above explanation. As shown in Figure 3b, LCER0 exhibited a broad diffuse peak at 15°–25°, suggesting the amorphous structure formed within the cured network.^[^ ^39^ ^]^ As the proportion of LCE increased, diffraction peaks at ≈19.5°, 22.2°, and 27.6° were gradually visible and getting stronger, suggesting microscopically ordered crystal‐like arrangement was present. The absence of signals at 5°–10° indicated they were nematic liquid crystalline phase.^[^ ^40^ ^]^ Meanwhile, the diffraction peaks at 27.6° and 28.3° confirmed the existence of π–π stacking within the cross‐linking network. The π–π interactions not only contributed to the alignment and stability between molecules but also provided strong interactions between molecular chains, enabling heat to be conducted more efficiently between the stacking planes.^[^ ^40^, ^41^ ^]^ Based on the Scherrer formula ($D=\frac{k\lambda }{B\cos\theta }$, where *k* = 0.89; *λ* = 0.154 nm, here *λ* is the wavelength; *B* is the half‐peak height of the diffraction peaks; *θ* is the diffraction angle),^[^ ^42^ ^]^ the grain sizes (*D*) of LCER2, LCER3, and LCER4 were calculated to be increased from 4.27 to 10.37 and to 17.81 nm, which was consistent with the increasing fraction of LCE. According to the Bragg formula (2d sin θ =  *n*λ; where *λ* = 0.154 nm, here *λ* is the wavelength; *θ* is the diffraction angle),^[^ ^43^ ^]^ the interplanar spacings (*d*) were 0.44, 0.39, and 0.32 nm for LCER3, and 0.46, 0.41, and 0.33 nm for LCER4. This is because some liquid crystal units would first self‐assemble into crystal nucleus through π–π stacking. The remaining liquid crystals further grew around the crystal nucleus to eventually form spherulites. These spherulites were distributed as ordered microdomains within the disordered cross‐linked network. As the number and size of the spherulites increased, the grain size increased and eventually formed continuous thermal conduction pathways connected within the cross‐linked network to increase thermal conductivity.

Small‐angle X‐ray scattering (SAXS) analyses (Figure S3, Supporting Information) were further employed to investigate the microstructures of LCER vitrimers. Clearly, LCER0 showed no scattering peaks, indicating an amorphous cross‐linked network. In contrast, strong scattering peaks at *q* = 0.05–0.25 Å^−1^ appeared with increasing the amount of LCE, demonstrating electron density difference existing in the cured network. This represented the presence of mesogens aggregation and microphase separation. The distances between the domains (*L*) of LCER2, LCER3, and LCER4 were further calculated according to equation $L=\frac{2\pi }{q}$,^[^ ^44^ ^]^ which were 13.28, 8.75, and 6.87 nm, respectively, suggesting the ordered periodic spacing was shortening. This is attributed to the fact that the number and size of crystalline domains were growing as increasing the content of LCE, thus resulting in a denser distribution of grain domains. The POM images in Figure 3c revealed no birefringence in LCER0, further indicating a disordered state in the cross‐linking network. However, bright yellow spots of birefringence gradually appeared with increasing the LCE until spreading over the whole visual area for LCER4, confirming the presence of crystalline domains within the cured network. The analyses of XRD, SAXS, and POM were consistent. These results confirmed the order orientation was enhanced within the cured network as increasing the biphenyl mesogens, which increased the MFP of phonons and suppressed phonon scattering, thus improving the thermal conductivity as shown in Figure 3d.

### 2.3 Thermal Resistance and Mechanical Properties of LCER Vitrimers

**Figure 4:**
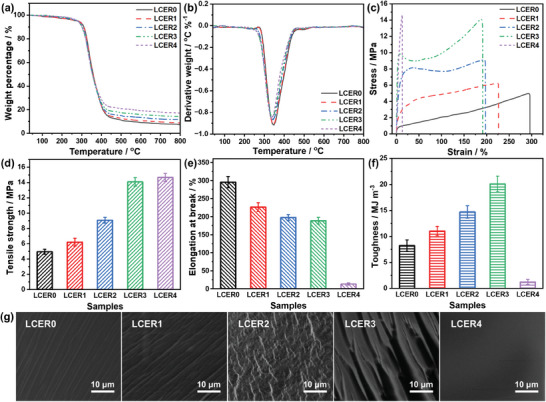
TGA (a), DTG (b), and stress‐strain curves (c), tensile strength (d), elongation at break (e), toughness (f), and SEM images of tensile fracture section (g) of LCER0‐4.

The thermal resistance of LCER vitrimers was explored by thermogravimetric analysis (TGA). As shown in **Figure** **4**a, only less than 5% of weight was lost when the temperature reached 278 °C, indicating high thermal resistance of LCER vitrimers. The differential thermal gravity (DTG)^[^ ^45^ ^]^ curves in Figure 4b exhibited rapid decomposition between 278 and 410 °C, which was attributed to the breakage of disulfide bonds with low bond energies and destruction of cross‐linking network. It is worth mentioning that the carbon residue rate increased to 17.2% for LCER4 from 7.5% for LCER0 (Table S3, Supporting Information). This can be ascribed to the fact that biphenyl units can enhance the thermal stability of the polymer during the thermal treatment process while helping to retain a higher proportion of carbon residue.

The mechanical properties of LCER vitrimers were investigated employing a uniaxial tensile test and the corresponding stress‐strain curves were displayed in Figure 4c. As the fraction of LCE increased, LCER vitrimer was transformed from soft and weak of LCER0 into hard and tough of LCER3. For instance, the elongation at break decreased from 295.4% to 188.7% while the tensile strength increased from 4.9 to 14.1 MPa and the toughness increased from 8.2 to 20.1 MJ m^−3^. However, by further increasing the content of LCE (LCER4), although the vitrimer showed a relatively high tensile stress of 14.7 MPa, the elongation at break and toughness decreased sharply to 13.2% and 1.2 MJ m^−3^, respectively (Figure 4d–f). The reason for the increase of tensile stress and toughness by increasing LCE can be mainly ascribed to the π–π interactions of liquid crystalline units, which greatly increased the regularity of the cured network and was conducive to stress resistance. Besides, the highly ordered arrangement of the biphenyl mesogens enhanced the rigidity of the cross‐linking network, resulting in the improvement of toughness. However, under the circumstance of cured network without any addition of E‐51 (LCER4), the molecular chains were tightly packed in the microscopic level to form large crystalline domains which were difficult to move under external force.^[^ ^46^ ^]^ Therefore, the elongation at break and toughness significantly declined for LCER4. The tensile fracture of LCER vitrimers was further examined by scanning electron microscope (SEM, Figure 4g). It was found that silver streaks were observed for all the tensile sections of LCER0, LCER1, LCER2, and LCER3, which was produced by the high degree of orientation of polymer chains along the direction of tensile stress that absorbed energy. The SEM results of the fracture morphology for these vitrimers suggested that the fracture modes were ductile fracture. The silver streaks of LCER0 were highly oriented and developed regularly in one direction, indicating that the cured network was uniform and isotropic. In contrast, the silver streaks became unorderly as increasing LCE, indicating that the degree of homogeneity was affected by the locally ordered region formed by LCE. Meanwhile, the silver streaks of LCER3 evolved significantly wider. This was because the molecular chains at the interface between the oriented silver streaks and matrix were continuously transferred into silver streaks under stress. The stress fracture of LCER4 displayed a relatively smooth and clean morphology, indicating brittle fracture characteristics. These results were essentially in agreement with the mechanical properties. Considering the optimal thermal conductivity and mechanical properties, LCER3 vitrimer was selected for the following study.

### 2.4 Reprocessing and Shape Memory Behaviors of LCER Vitrimers

**Figure 5:**
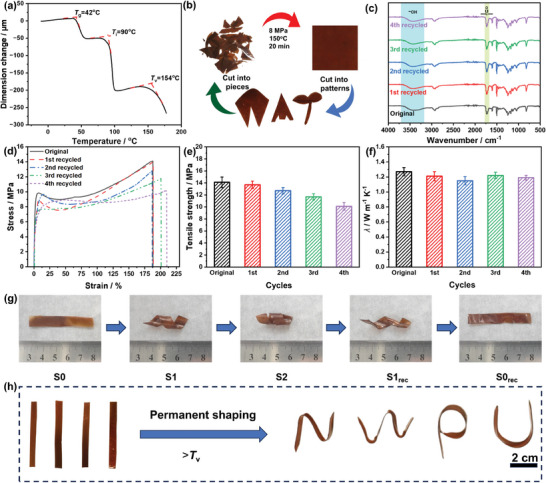
TMA curve (a), schematic of reprocessing (b), FT‐IR spectra (c), stress‐strain curves (d), tensile strength (e), and *λ* values (f) after cyclic reprocessing of LCER vitrimer; triple shape memory behavior (g) and 3D permanent shape reconstruction (h) of LCER vitrimer.

The liquid crystalline groups and dynamic covalent bonds endow LCER vitrimer with shape memory and reprocessing properties, respectively. As shown in the thermal mechanical analysis (TMA, **Figure** **5**a), the *T*_g_, *T*_i_, and dynamic covalent bond exchange temperatures (*T*_v_) of LCER vitrimer were found to be 42, 90, and 154 °C, respectively. The reprocessing property was investigated by cutting LCER vitrimer into pieces and remolding by hot pressing at 150 °C under 8 MPa pressure. As displayed in Figure 5b, the vitrimer pieces were successfully reprocessed into integrated specimens within 20 min. Similar characteristic peaks were observed from the FT‐IR spectra of the reprocessed vitrimer even after four cycles of reprocessing (Figure 5c), indicating the chemical structure remained intact. Notably, the LCER vitrimer still exhibited hard and tough mechanical properties after four cycles of reprocessing by showing a 72% retained tensile strength of 10.2 MPa to the original value (Figure 5d,e). Meanwhile, the reprocessed vitrimer still maintained 94% of its original *λ* after four cycles of reprocessing (Figure 5f).

In addition, due to the unique thermotropic driving response of liquid crystalline units, the temporary shape of LCER vitrimer produced by external forces could be fixed or restored by changing temperature so as to exhibit triple shape memory behavior. As shown in Figure 5g, the original long strip sample (S0) of LCER vitrimer was deformed into a helical shape (S1) by an external force at 120 °C (>*T*_i_) owing to the fully released segmental motion. The temporary helical shape could be fixed by removing the external force after the temperature was reduced to 60 °C as the crystalline region was frozen. The other helical shape (S2) was obtained by external force at 20 °C, which returned to the previous helical shape (S1_rec_) once reheated to 60 °C under no external force. This is because the crystalline structure of LCER vitrimer remained intact and only segmental motion proceeded at temperature of higher than *T*_g_ and lower than *T*_i_ (see Figure S4 and description, Supporting Information), enabling the shape memory behavior of LCER vitrimer. By further reheating to 120 °C, the vitrimer returned to its original strip shape (S0_rec_) under no external force. The dynamic covalent bond exchange can be initiated when the temperature is above *T*_v_ so as to rearrange the topology of the cross‐linked network to different shapes. As exhibited in Figure 5h, four long strip samples of LCER vitrimer could be respectively reconfigured into different letter shapes (NWPU), which could still maintain the shape even when reheated above the *T*_v_ without external force. The above results suggested that LCER vitrimer had excellent reprocessing and shape memory performance.

### 2.5 Photothermal Effect Induced Programming, Self‐Healing, and Welding of LCER Vitrimers

**Figure 6:**
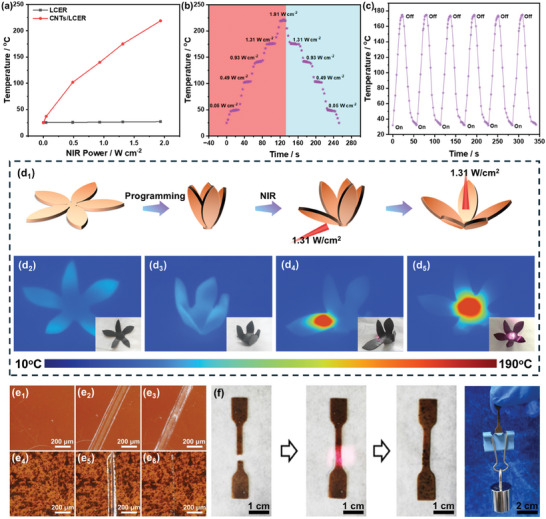
The surface temperature of LCER and CNTs/LCER vitrimers under different NIR (808 nm) irradiation with densities of 0.05, 0.49, 0.93, 1.31, and 1.91 W cm^−2^ (a); surface temperature upon changed NIR (808 nm) densities (b); heating stability and repeatability of CNTs/LCER vitrimer upon the repeated NIR (808 nm) density of 1.31 W cm^−2^ (c); corresponding thermal infrared images (inset images are digital photos of CNTs/LCER vitrimers) during the shape memory process of the accurate remote controlled deformation recovery using NIR (808 nm) of 1.31 W cm^−2^ (d_1_–d_5_); microscopic images of the surface for unscratched (e_1_,e_4_), scratched (e_2_,e_5_), and welded (e_3_,e_6_) LCER (top) and CNTs/LCER (bottom) vitrimers; process of NIR‐welding and welded CNTs/LCER vitrimer loaded with a 50 g weight (f).

Due to the photothermal effect, CNTs are usually harnessed in constructing photo responsive materials. To endow photothermal effect, 0.5 wt% CNTs were introduced into the cross‐linking network of LCER vitrimers (the thermal conductivity was not affected as shown in Figure S5, Supporting Information). By irradiating the LCER vitrimer without CNTs using an 808 nm NIR light with different optical powers (0.05, 0.49, 0.93, 1.31, and 1.91 W cm^−2^), the surface temperature remained unchanged even after 40 s of irradiation. In contrast, the surface temperature of LCER vitrimer with CNTs (CNTs/LCER) increased rapidly to 37, 102, 140, 175, and 219 °C, respectively, which can be attributed to the excellent photothermal conversion ability of CNTs (**Figure** **6**a). Additionally, the surface temperature of CNTs/LCER vitrimer displayed rapid responsiveness without obvious hysteresis by step increasing or decreasing the optical power density (Figure 6b). In order to ensure that the operating temperature of LCER is only slightly higher than *T*_v_, 1.31 W cm^−2^ was selected as the optical power density for the following study. The CNTs/LCER vitrimer exhibited excellent cycling performance of photothermal conversion when irradiated by the NIR light. As shown in Figure 6c, the surface temperature of the CNTs/LCER vitrimer increased rapidly to 173.8–175.1 °C when the NIR light was turned on and decreased instantly to 31.9–32.5 °C when the NIR light was turned off. It can be concluded that CNTs/LCER vitrimer displayed stable photothermal conversion performance during the multiple cycles while being able to realize “on” and “off” switch on demand.

The photothermal conversion performance of CNTs/LCER vitrimer could be employed for light‐induced programming of the vitrimer by precise control (Figure 6d1). For instance, the CNTs/LCER vitrimer was first cut into a flower‐like sample (Figure 6d2) and programmed to a flower‐shaped bud with petals closed by applying certain temperature and pressure (Figure 6d3). Next, the NIR light was positioned on the base of one petal, which was precisely irradiated to open while other petals maintained their programmed closed shape (Figure 6d4, Movie S1, Supporting Information). Furthermore, when the root of the flower was irradiated by NIR light, all the petals opened up and returned to the original shape (Figure 6d5, Movie S2, Supporting Information). The infrared thermal images showed that the temperature of NIR‐irradiated area was able to increase from 28 to 165 °C within 20 s, further confirming the efficient photothermal conversion performance of CNTs/LCER vitrimer.

The photo‐thermal induced self‐healing behavior of the CNTs/LCER vitrimer (Figure 6e4–e6) was investigated by a comparative study with the LCER vitrimer without CNTs (Figure 6e1–e3). For instance, both the surfaces of the two different types of vitrimers were scratched to a crack and then irradiated with a NIR light of 1.31 W cm^−2^. Obviously, the scratch on the surface of LCER vitrimer without CNTs remained unhealed due to the absence of photothermal effect after NIR light irradiation (Figure 6e3). In contrast, the crack of the CNTs/LCER vitrimer was rapidly healed within 10 s after NIR light irradiation (Figure 6e6), which was attributed to the rapid increase of temperature to *T*_v_ in the NIR irradiated region and activated the dynamic exchange of disulfide and ester bonds. To further evaluate the welding capability, the CNTs/LCER vitrimer strip was pulled off and welded by irradiating NIR light. As shown in Figure 6f, the fracture of the strip was successfully welded after 20 s of NIR light irradiation. The welded CNTs/LCER vitrimer could still hold a 50 g weight, which was 500 times its own weight.

### 2.6 Closed‐Loop Recycling and Degradation of CNTs/LCER Vitrimer

**Figure 7:**
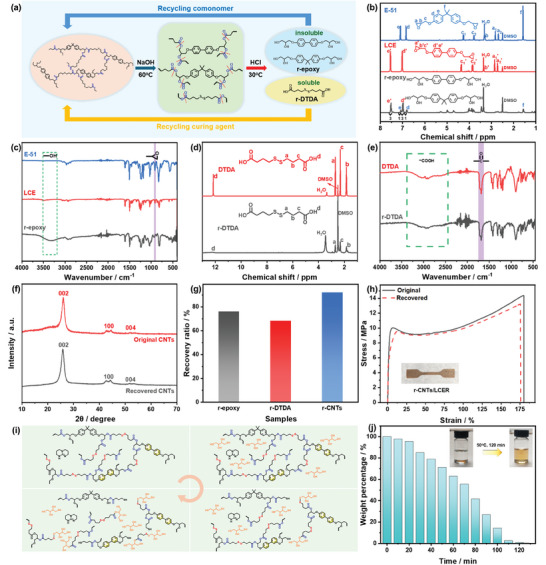
Schematic diagram of closed‐loop recycling of CNTs/LCER vitrimer (a); ^1^H NMR (b) and FT‐IR (c) spectra of r‐epoxy; ^1^H NMR (d) and FT‐IR (e) spectra of DTDA and r‐DTDA; XRD curves of original and recycled CNTs (f); recycling efficiencies of r‐epoxy, r‐DTDA, and r‐CNTs (g); stress‐strain curves of original and recycled CNTs/LCER vitrimer, inset showing the digital image of recycled CNTs/LCER vitrimer (h); proposed degradation process of LCER vitrimer in the presence of DTT (i); degradation kinetics of CNTs/LCER vitrimer in DTT/DCM at 50 °C, inset images showing the sample before and after degradation (j).

The recyclability of the CNTs/LCER vitrimer was then explored applying a depolymerization strategy. The CNTs/LCER vitrimer was subjected to the aqueous processing sequence (depolymerize, separate, precipitate, and extract) as described in experimental section, which allowed E‐51, LCE, DTDA, and CNTs to be recovered. The depolymerization of LCER vitrimer (**Figure** **7**a) was monitored using ^1^H nuclear magnetic resonance (NMR) spectra. As shown in Figure 7b, the recovered epoxy (r‐epoxy) monomer contained LCE and E‐51 as characteristic peaks corresponding to the protons in their benzene rings were observed. Besides, the integration ratio of the respective peaks corresponding to LCE and E‐51 was 3:1, which was in agreement with the original formulation. It needs to be noted that the characteristic peaks of protons in epoxy groups of LCE and E‐51 were not visible anymore, which was caused by the ring opening of epoxy groups once cured and replaced by the hydroxyl groups when depolymerized. This can also be verified by the FT‐IR spectrum of r‐epoxy (Figure 7c), in which the characteristic peak of epoxy group at 913 cm^−1^ disappeared while the hydroxyl group appeared at 3300 cm^−1^. Meanwhile, the recovered DTDA (r‐DTDA) demonstrated identical ^1^H NMR (Figure 7d) and FT‐IR (Figure 7e) spectra to the original one. Additionally, the XRD curve of the recovered CNTs (r‐CNTs, Figure 7f) was consistent with the original CNTs, indicating that the recycling process would not destroy the crystal structure of CNTs. The recovery ratio of epoxy monomer, DTDA, and CNTs was found to be 76.1, 68.4, and 92.1% (Figure 7g), respectively, indicating relatively high recycling efficiencies. The recycled epoxy monomer and DTDA were combined with CNTs to prepare recycled CNTs/LCER vitrimer. It exhibited 92% recovery of tensile strength (Figure 7h) and 90% recovery of thermal conductivity (Figure S6a, Supporting Information), respectively, while still possessing excellent photothermal effect (Figure S6b, Supporting Information). The above results proved that CNTs/LCER vitrimer possessed closed‐loop recycling performance.

Owing to the dynamic exchange reactions of disulfide and ester bonds, the CNTs/LCER vitrimer could be readily degraded by dithiothreitol (DTT).^[^ ^46^ ^]^ The degradation kinetics of the CNTs/LCER network was investigated in a DCM solution of DTT (20 mmol L^−1^) at 50 °C by monitoring the residual mass of the sample. As shown in Figure 7i, the degradation could be mainly divided into the following stages. First, DTT molecules and DCM diffused into the cross‐linked network, so that the LCER network started to swell. Then, the thiol‐disulfide and ester‐exchange reactions begin to occur with the presence of DTT, leading to partial degradation of the network. Finally, the network is completely degraded into small molecular fragments and dissolved in the DTT/DCM solution. Owing to the flexible cross‐linked network, DTT exhibited a fast diffusion rate into the network, resulting in rapid degradation.^[^ ^25^, ^47^ ^]^ As displayed in Figure 7j, the vitrimer sample went through a swelling period for 20 min, after which the sample started degrading, and degraded completely after 120 min. These results indicated that CNTs/LCER vitrimer displayed excellent degradability.

## 3 Conclusion

In conclusion, highly thermally conductive liquid crystalline epoxy resin (LCER) vitrimers with reprocessing and multiple shape memory behavior were prepared by combining the synergistic effects of liquid crystals and dynamic covalent bonds. The results showed that the content of liquid crystals effectively modulated the thermal conductivity and mechanical properties of LCER vitrimer. The in‐plane thermal conductivity of LCER vitrimer increased from 0.61 W m^−1^ K^−1^ of pure E‐51 vitrimer to 1.27 W m^−1^ K^−1^ by introducing 75 mol% of liquid crystalline epoxy. Meanwhile, LCER vitrimer demonstrated significantly enhanced mechanical properties, with a tensile strength of 14.1 MPa and a toughness of 20.1 MJ m^−3^, which were 188% and 145% higher than those of pure E‐51 vitrimer (4.9 MPa and 8.2 MJ m^−3^), respectively. Notably, LCER vitrimer showed excellent reprocessing performance by maintaining 94% and 72% of the original thermal conductivity and tensile strength after four cycles of reprocessing. Additionally, LCER vitrimer with 0.5 wt% CNTs exhibited stable photothermal conversion performance, enabling “on” and “off” switch on demand. The CNTs/LCER vitrimer also showed precise NIR light‐induced actuation by controlling the position of the incident light. Besides, the CNTs/LCER vitrimer exhibited shape memory, self‐repairing, and self‐welding capabilities under NIR light due to the photothermal effect, along with closed‐loop recycling and rapid degradation properties. This work not only broadens the applications of epoxy resins, providing insights for the design and fabrication of multifunctional epoxy resin vitrimers, such as flexible heat spreader, personal thermal management, flexible wearable electronics, and soft actuators, but also contributes to reducing environmental pollution and advancing sustainability.

## 4 Experimental Section

### Synthesis of LCE

The synthetic approach of LCE was demonstrated in Scheme S1 (Supporting Information). First, 4,4′‐dihydroxybiphenyl (10 g, 54 mmol), epichlorohydrin (42.5 mL, 64.2 mmol), isopropanol (33 mL), and deionized water (9.5 mL) was introduced into a three‐necked flask and mixed evenly, which was then placed in an oil bath at 90 °C for 30 min. Then, 9.5 mL of 15 wt% NaOH aqueous solution was added drop by drop over a period of 1 h. The reaction was continued for 1 h followed by the drop‐wise addition of another 9.5 mL of 15 wt% NaOH aqueous solution. The reaction mixture was cooled to room temperature after 1 more hour. The precipitated solid was filtered and washed with water, ethanol‐water mixture (1/1, vol/vol), and ethanol in sequence. The obtained white solid was dried in an oven of 60 °C overnight to afford LCE with an 85.5% yield, which was characterized by ^1^H (Figure S1a, Supporting Information) and ^13^C (Figure S1b, Supporting Information) NMR spectra, FT‐IR spectrum (Figure S1c, Supporting Information), and XRD (Figure S1d, Supporting Information).

### Preparation of LCER Vitrimers and CNTs/LCER Vitrimer

LCER vitrimers were prepared by a hot press molding process as follows. First, a certain amount of E‐51 was added to a polytetrafluoroethylene beaker and heated to be transparent at 150 °C. Then, an appropriate amount of LCE was added and stirred to be dissolved. DTDA was then added slowly with continuous stirring until the mixture was homogeneous, followed by addition of 1,5,7‐triazabicyclo[4.4.0]dec‐5‐ene (TBD) as the transesterification catalyst. The mixture was stirred evenly and quickly transferred to the between of two metal plates covered with a polyimide film and cured for 4 h at 150 °C under the pressure of 8 MPa to generate LCER vitrimers. The ratio of E‐51 to LCE is shown in Table S1 (Supporting Information) and the LCER vitrimers were noted as LCER0, LCER1, LCER2, LCER3, and LCER4, respectively. CNTs/LCER vitrimer was prepared in the same way with LCER vitrimers except 0.5 wt% of CNTs was added after dissolving of LCE.

### Closed‐Loop Recycling of CNTs/LCER Vitrimer

The CNTs/LCER vitrimer was placed in a 30 wt% NaOH aqueous solution and stirred at 60 °C for 8 h to completely decompose the LCER vitrimer into monomers and release the CNTs. The mixture was then acidified to pH = 5 with 1 mol/L hydrochloric acid to afford an aqueous solution of DTDA and NaCl, and insoluble solid of CNTs and degraded LCE and E‐51, which were insoluble in chloroform. The insoluble solid was dispersed in chloroform to stand for 12 h to separate the recovered LCE and E‐51 (r‐epoxy) from CNTs based on their density difference. The aqueous solution of DTDA and NaCl was dried to obtain the solid of DTDA and NaCl, which were then dispersed in ethanol to separate insoluble NaCl. The DTDA‐ethanol solution was then dried using rotary evaporation to afford recovered DTDA (r‐DTDA). The recycled LCE, E‐51, DTDA, and CNTs were applied to prepare recycled CNTs/LCER (r‐CNTs/LCER) vitrimer following the same procedure in section of *Preparation of LCER vitrimers and CNTs/LCER vitrimer*.

### Statistical Analyses

The thermal conductivity, tensile strength, elongation at break, and toughness were presented as the means ± standard deviation (SD).

## Conflict of Interest

The authors declare no conflict of interest.

## Supporting information



Supporting Information

Supplemental Movie 1

Supplemental Movie 2

## Data Availability

The data that support the findings of this study are available in the supplementary material of this article.
