# Supplementary material for: Highly Thermally Conductive Liquid Crystalline Epoxy Resin Vitrimers with Reconfigurable, Shape‐Memory, Photo‐Thermal, and Closed‐Loop Recycling Performance
Source: Adv Sci (Weinh). 2024 Nov 22;12(3):2410362. doi: 10.1002/advs.202410362 (PMC11744650; doi:10.1002/advs.202410362)
Supplement: Supplementary file 1 — Supporting Information [file ADVS-12-2410362-s003.docx]

Supporting Information

Highly Thermally Conductive Liquid Crystalline Epoxy Resin Vitrimers with Reconfigurable, Shape-Memory, Photo-Thermal, and Closed-Loop Recycling Performance

Fengyuan Zhang, Junliang Zhang*, Kuan Zhang, Xiao Zhong, Mukun He, Hua Qiu, Junwei Gu*

F. Zhang, J. Zhang, K. Zhang, X. Zhong, M. He, H. Qiu, J. Gu

Shaanxi Key Laboratory of Macromolecular Science and Technology, School of Chemistry and Chemical Engineering, Northwestern Polytechnical University, Xi’an, Shaanxi, 710072, P. R. China
E-mail: junliang.zhang@nwpu.edu.cn (J. Zhang); gjw@nwpu.edu.cn & nwpugjw@163.com (J. Gu).

**S1. Experimental section**

**S1.1. Chemicals and Materials**

4,4'-dihydroxybiphenyl (BP, 97%) and 1,5,7-triazabicyclo[4.4.0]dec-5-ene (TBD, 97%) were purchased from Aladdin Shanghai Co., Ltd. (Shanghai, China). Epichlorohydrin (ECH, 99.5%), isopropanol (IPA, 99.5%), and carbon nanotubes (CNTs, inside diameter: 5-10 nm, outside diameter: 10-20 nm, length: 30-100 μm, >95%) were purchased from Shanghai Macklin Biochemical Co., Ltd (Shanghai, China). 4,4'- Dithiodibutyric acid (DTDA, 95%) was purchased from Merck KGaA, Germany. Sodium hydroxide (NaOH, analytical grade) and anhydrous ethanol (EtOH, analytical grade) were purchased from Guangdong Guanghua Science and Technology Co., Ltd. Bisphenol A epoxy resin (E-51, chemically pure) was purchased from Laizhou Baichen Insulation Material Co., Ltd.


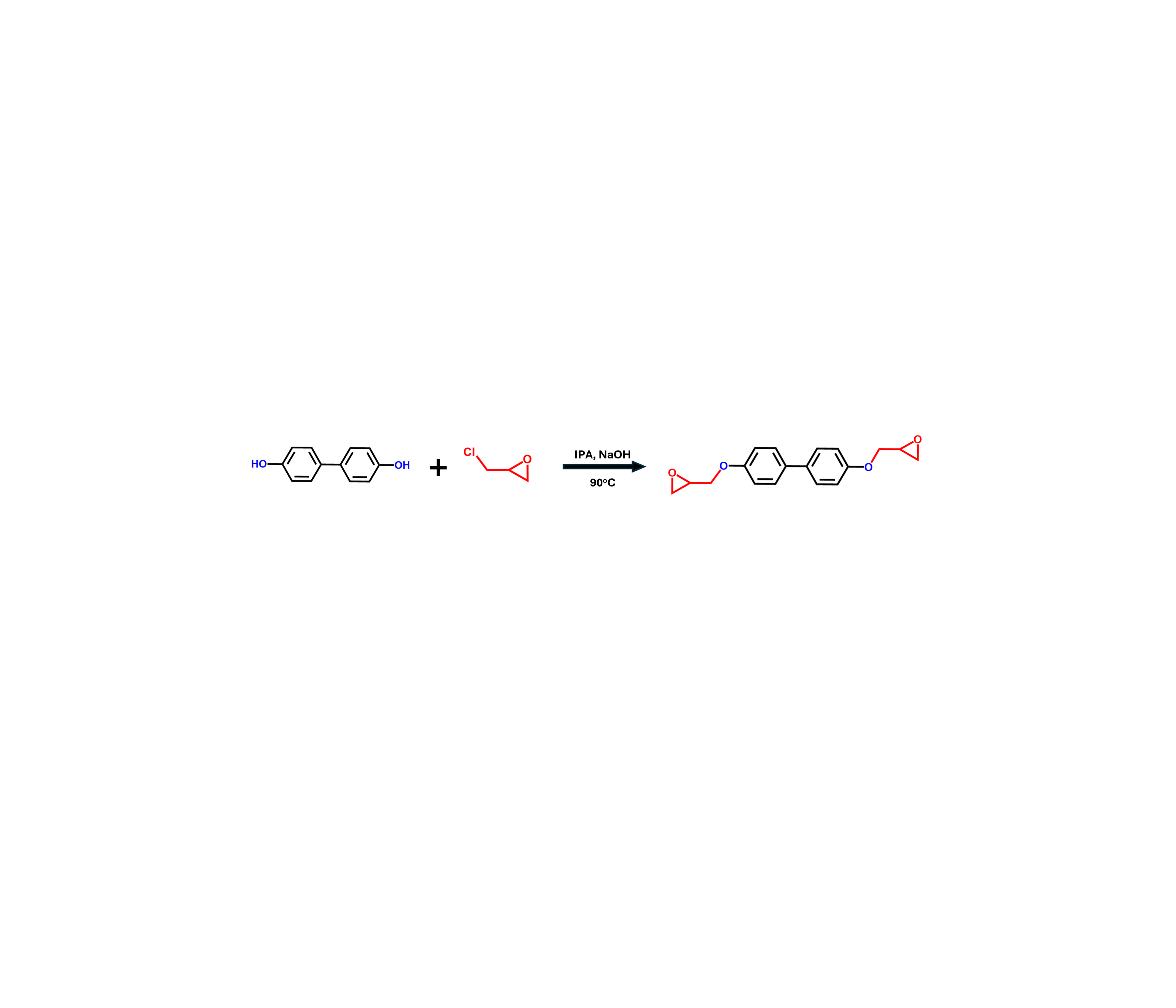


**Scheme S1.** Synthetic route of LCE

**Table S1.** Material feeding ratios of LCER vitrimers

|  | E-51 / mmol | LCE / mmol | DTDA / mmol | TBD / mmol |
| --- | --- | --- | --- | --- |
| LCER0 | 10 | 0 | 10 | 0.25 |
| LCER1 | 7.5 | 2.5 | 10 | 0.25 |
| LEER2 | 5 | 5 | 10 | 0.25 |
| LCER3 | 2.5 | 7.5 | 10 | 0.25 |
| LCER4 | 0 | 10 | 10 | 0.25 |

n (DTDA) = n (E-51) + n (LCE), n (TBD) = 2.5% n (DTDA)

**S1.2. Characterization**

The molecular structure of the samples was characterized by proton and carbon nuclear magnetic resonance (^1^H NMR and ^13^C NMR, Bruker Avance 400 MHz, Bruker, Germany) spectroscopy. Deuterated chloroform or DMSO were used as solvents with tetramethylsilane (TMS) as an internal standard.

Fourier transform infrared (FT-IR, Bruker Tensor II, Bruker, Germany) spectroscopy was used to characterize the functional groups of the samples. The characterization method was attenuated total internal reflectance (ATR) with the testing range of 400-4000 cm^-1^.

X-ray diffraction (XRD) patterns of the samples were tested using an XRD (D8 Advance, Bruker, Germany) machine equipped with a Cu-targeted radiation source. The scanning speed was 10^o^/min with the diffraction angle of 2θ = 5^o^ to 70^o^. The step size was 0.02^o^/step.

Small-angle X-ray scattering (SAXS) curves of the samples were tested using a SAXS machine (Xeuss 3.0, France) equipped with a Cu-Kα targeted radiation source. The distance from the sample to the detector was 300 nm. The scattering angle of 2θ was from 0.3^o^ to 10^o^.

The liquid crystalline and curing behaviors of the samples were characterized using a differential scanning calorimetry (DSC, DSC1, Mettler-Toledo, Switzerland). The heating rate was 10^o^C/min and the atmosphere was nitrogen.

Thermogravimetric analysis (TGA, STA449F3, Germany) was used to investigate the thermal properties of the samples over a temperature range of 30 to 800^o^C with a heating rate of 10^o^C/min under a nitrogen atmosphere.

A thermo-mechanical analyzer (Discovery TMA 450, China) was used to test the dynamic covalent bond exchange temperatures of the sample in the range of 0-200^o^C. The sample size for the TMA was 5×5×4 mm. The heating rate was 10^o^C/min and the atmosphere was nitrogen.

A hot-stage polarizing optical microscope (POM, WMP-6880, Shanghai Wumo Optical Instrument, China) was used to observe the liquid crystalline behavior of the samples with a heating rate of 10^o^C/min.

A scanning electron microscope (SEM, Verios G4, FEI, USA) was used to observe the morphology of tensile fracture of LCERs. The samples were sprayed with gold before testing.

Infrared thermal images of the samples were captured using a Ti300 infrared thermal imager (Fluke, USA). The hot stage was preheated to 80^o^C.

Calculation of gel content: Samples (~50 mg, m0) were respectively immersed in water, hydrochloric acid, methanol, ethanol, dichloromethane, chloroform, and acetone for 20 hours at room temperature. Each sample was then dried in a vacuum oven at 60^o^C to get the dried weight (m1). The gel content was calculated using equation (S1).

$Gel content \left（ \% \right）=\frac{m1}{m0}\times100\%$ (S1)

Calculation of swelling ratio: Samples (~50 mg, m0) were respectively immersed in water, hydrochloric acid, methanol, ethanol, dichloromethane, chloroform, and acetone for two weeks. After taking out of the solvent, excess solvent on the surface was removed with filter paper and the weight of the swollen sample was noted as m1. The swelling ratio was calculated using Equation (S2).

$Swelling ratio \left（ \% \right）=\frac{m1-m0}{m0}\times100\%$ (S2)

The thermal conductivity was tested using a thermal constant analyzer (TPS2200, Hot Disk, Sweden) using the flat plate method according to the standard ISO 22007-2:2008 at 25^o^C. The radius and thickness of the specimen were 15±1 mm and 350±20 μm, respectively. Measurements were repeated three times and the average result of these three measurements was used for the thermal conductivity of each sample.

The tensile strength and elongation at break of the specimens were measured using an electronic universal testing machine from Dongguan Sitai Instruments Co., Ltd. The tensile rate was 20 mm/min. The dimensions of the specimen were as follows: length =35 mm, width of the end =6±0.5 mm, length of the narrow part =12±0.5 mm, width of the narrow part =2±0.1 mm, and the radius of the transition edge =3±0.1 mm. Measurements were repeated three times and the average result of these three measurements was used for each sample.

A near-infrared laser module (FU808MLKJ-F3G, China) was used to test the photo-thermal properties of the samples. A laser power meter (HWLPM-Mini-10w, China) was used to measure the laser power density.


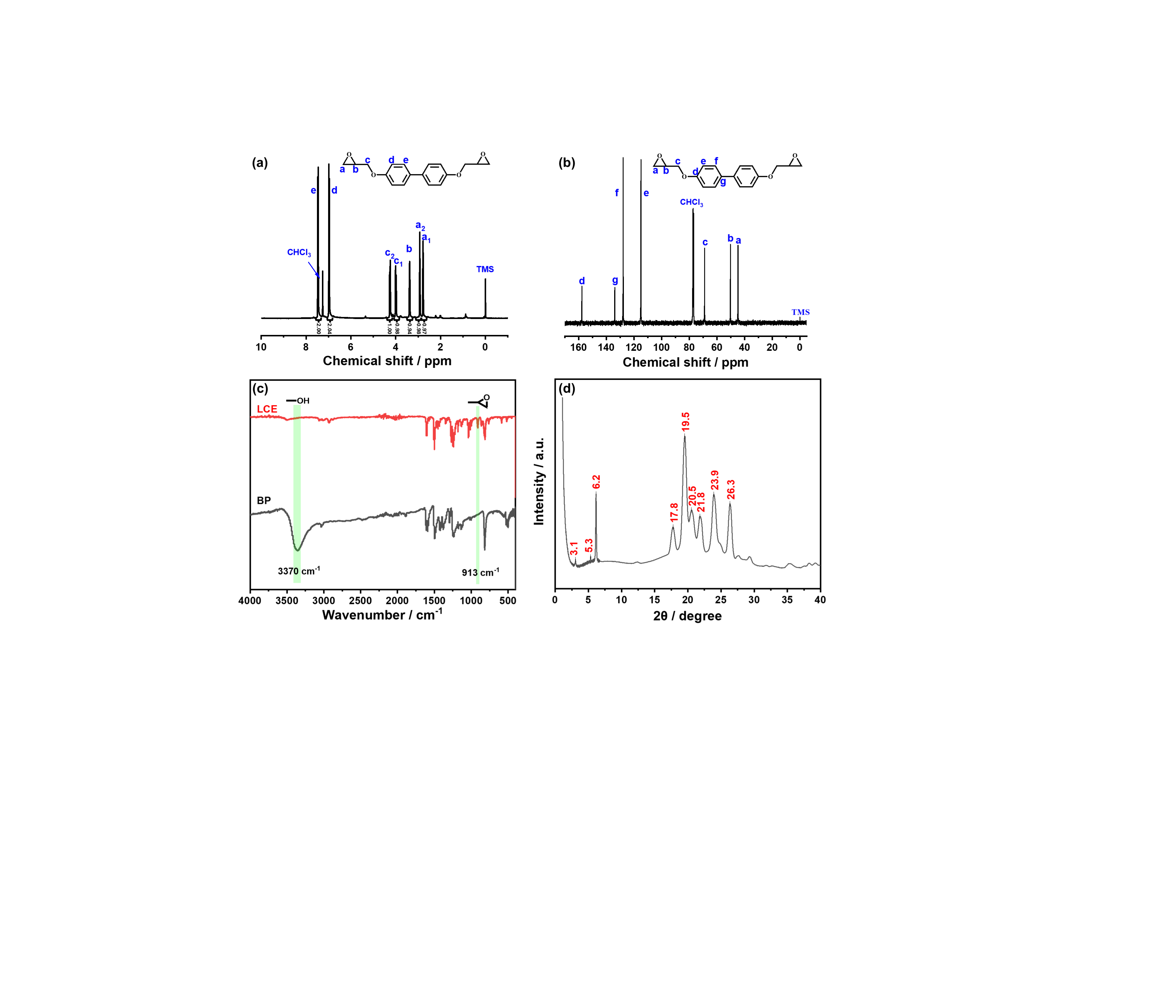


**Figure S1.** ^1^H NMR (a),^13^C NMR (b), FT-IR (c) spectra, and XRD curve (d) of LCE

^1^H NMR analysis of LCE (400 MHz, CDCl_3_, ppm): δ=7.48 (*d*, *J*= 8 Hz, –O–C*_6_***H*_4_***–C*_6_***H*_4_***–O–, **e**), δ=6.98 (*d*, *J*= 8 Hz, –O–C*_6_***H*_4_***–C*_6_***H*_4_***–O–, **d**), δ= 4.28 (*dt*, *J*=12, 4 Hz, –CH_2_(O)CH–C***H_2_***–CH–O–C_6_H_4_–, **c_2_**), δ=4.02 (*q*, *J*=8 Hz, –CH_2_(O)CH–C***H_2_***–CH–O–C_6_H_4_–, **c_1_**), δ=3.40~3.36 (*m*, –CH_2_(O)C***H***–CH_2_–CH–O–, **b**), δ=2.94~2.91 (*m*, –C***H_2_***(O)CH–CH_2_–CH–O–, **a_2_**), δ=2.79~2.77 (*m*, –C***H_2_***(O)CH–CH_2_–CH–O–, **a_1_**). ^13^C NMR analysis of LCE (100 MHz, CDCl_3_, ppm): δ=157.68, 133.86, 127.79, 114.94, 68.88, 50.21, and 44.74 ppm.


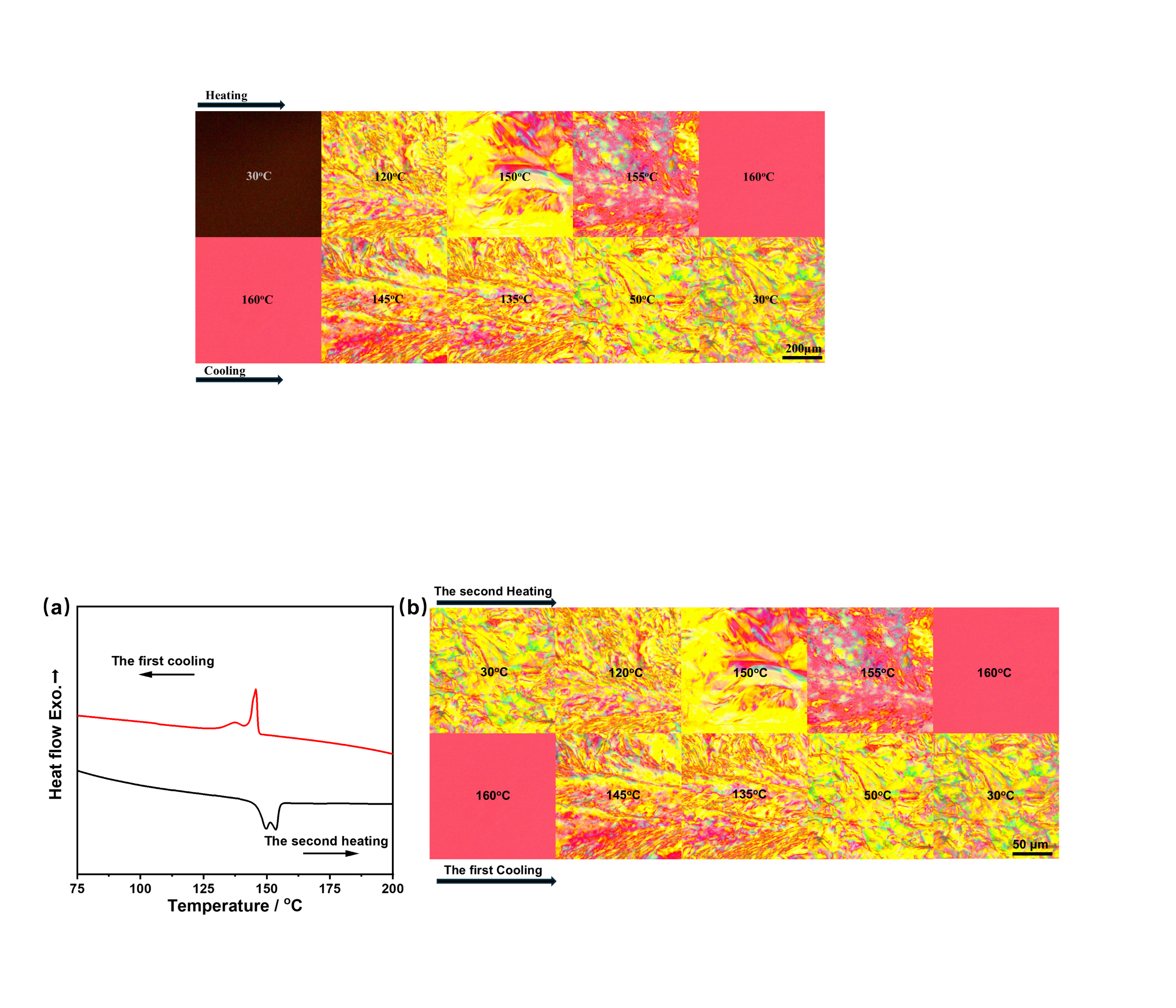


**Figure S2.** DSC curves (a) and POM images (b) of LCE during the heating and cooling steps

**Table S2.** Gel content and swelling ratio of LCER vitrimers

|  | Solvent | Gel content (%) | Swelling ratio (%) |
| --- | --- | --- | --- |
| LCER0 | H_2_O  HCl | 99.5 ± 0.4  99.2 ± 0.3 | 0.1 ± 0.01  0.1 ± 0.02 |
|  | MeOH | 98.5 ± 0.2 | 0.3 ± 0.01 |
|  | EtOH | 96.5 ± 0.3 | 0.2 ± 0.02 |
|  | CH_2_Cl_2_ | 99.2 ± 0.4 | 0.1 ± 0.02 |
|  | CHCl_3_ | 98.4 ± 0.3 | 0.2 ± 0.01 |
|  | Acetone | 99.3 ± 0.3 | 0.3 ± 0.01 |
| LCER1 | H_2_O  HCl | 99.2 ± 0.4  99.6 ± 0.3 | 0.1 ± 0.01  0.1 ± 0.03 |
|  | MeOH | 96.5 ± 0.1 | 0.4 ± 0.01 |
|  | EtOH | 99.1 ± 0.4 | 0.4 ± 0.01 |
|  | CH_2_Cl_2_ | 96.2 ± 0.2 | 0.6 ± 0.03 |
|  | CHCl_3_ | 99.2 ± 0.5 | 0.4 ± 0.01 |
|  | Acetone | 98.4 ± 0.5 | 0.5 ± 0.01 |
| LCER2 | H_2_O  HCl | 99.7 ± 0.4  99.5 ± 0.3 | 0.1 ± 0.02  0.1 ± 0.02 |
|  | MeOH | 99.2 ± 0.5 | 0.3 ± 0.01 |
|  | EtOH | 97.4 ± 0.4 | 0.2 ± 0.01 |
|  | CH_2_Cl_2_ | 99.1 ± 0.1 | 0.1 ± 0.02 |
|  | CHCl_3_ | 99.2 ± 0.4 | 0.5 ± 0.03 |
|  | Acetone | 96.1 ± 0.2 | 0.5 ± 0.03 |
| LCER3 | H_2_O  HCl | 99.6 ± 0.4  99.4 ± 0.4 | 0.1 ± 0.02  0.2 ± 0.01 |
|  | MeOH | 99.2 ± 0.5 | 0.4 ± 0.01 |
|  | EtOH | 99.4 ± 0.4 | 0.4 ± 0.01 |
|  | CH_2_Cl_2_ | 97.1 ± 0.1 | 0.3 ± 0.02 |
|  | CHCl_3_ | 99.2 ± 0.4 | 0.2 ± 0.03 |
|  | Acetone | 96.5 ± 0.3 | 0.4 ± 0.03 |
| LCER4 | H_2_O  HCl | 99.6 ± 0.4  99.5 ± 0.2 | 0.1 ± 0.01  0.1 ± 0.02 |
|  | MeOH | 99.2 ± 0.5 | 0.1 ± 0.01 |
|  | EtOH | 99.4 ± 0.4 | 0.4 ± 0.01 |
|  | CH_2_Cl_2_ | 99.1 ± 0.1 | 0.2 ± 0.02 |
|  | CHCl_3_ | 96.2 ± 0.4 | 0.1 ± 0.03 |
|  | Acetone | 99.2 ± 0.5 | 0.2 ± 0.02 |


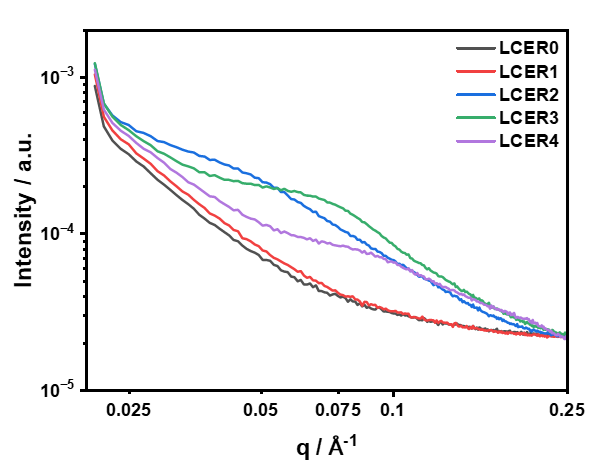


**Figure S3.** SAXS curves of LCER vitrimers

**Table S3.** Weight loss temperature and final carbon residue of LCER vitrimers after TGA test

| Sample | Temperature / ^o^C | | Carbon residue / % |
| --- | --- | --- | --- |
|  | 5 wt% | 30 wt% |  |
| LCER0 | 279.6 | 335.5 | 7.5 |
| LCER1 | 278.1 | 337.8 | 8.8 |
| LCER2 | 279.2 | 332.5 | 11.4 |
| LCER3  LCER4 | 280.7  279.7 | 331.6  331.0 | 14.2  17.2 |


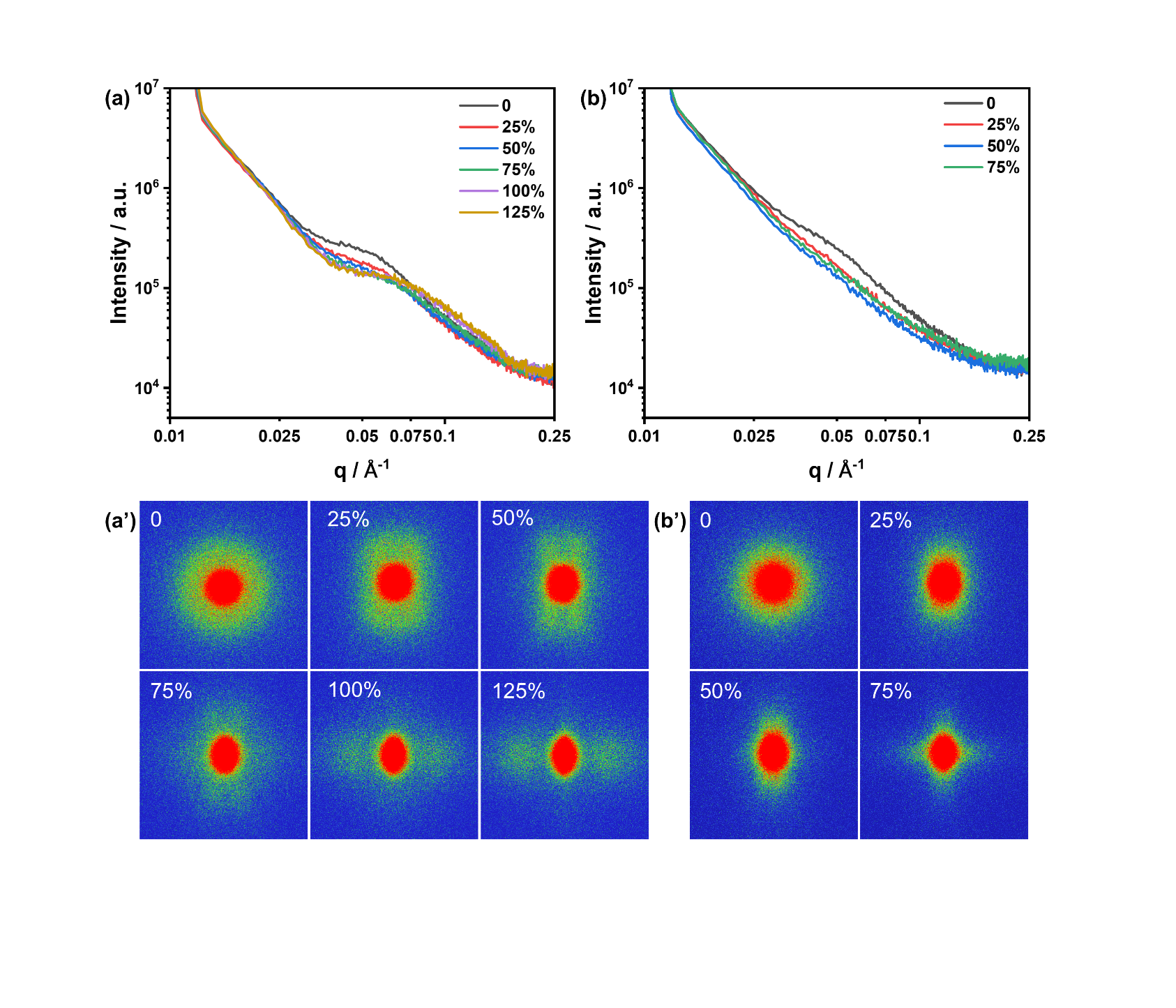


**Figure S4.** SAXS curves and 2D-SAXS images of LCER vitrimer under different in-situ stretching ratios at 53^o^C (a, a’) and 93^o^C (b, b’, the maximum stretching ratio was 75% at this temperature as the sample was too soft to be stretched further)

At 53°C (where *T*_g_<T<*T*_i_), the crystalline structure of LCER vitrimer should remain intact and only segmental motion proceeds. This can be verified by the increased q value as the stretching ratio increased, which indicated that the inter-spacing of the crystalline regions was decreasing (**Figure S4a**, **a’**). This is because the segments in the amorphous regions were also incorporated into the ordered regions under external force, leading to an increase in the ordered region area and a reduction in the periodic spacing. At 93°C (where *T*_i_ <T<*T*_v_), the crystalline regions of LCER vitrimer will be completely opened and the segmental motion will be fully released under external force. As shown in **Figure S4b**, **b’**, the scattering peaks disappeared completely with the increase of stretching ratio at this temperature, indicating a uniform electron cloud density and formation of homogeneity.

**
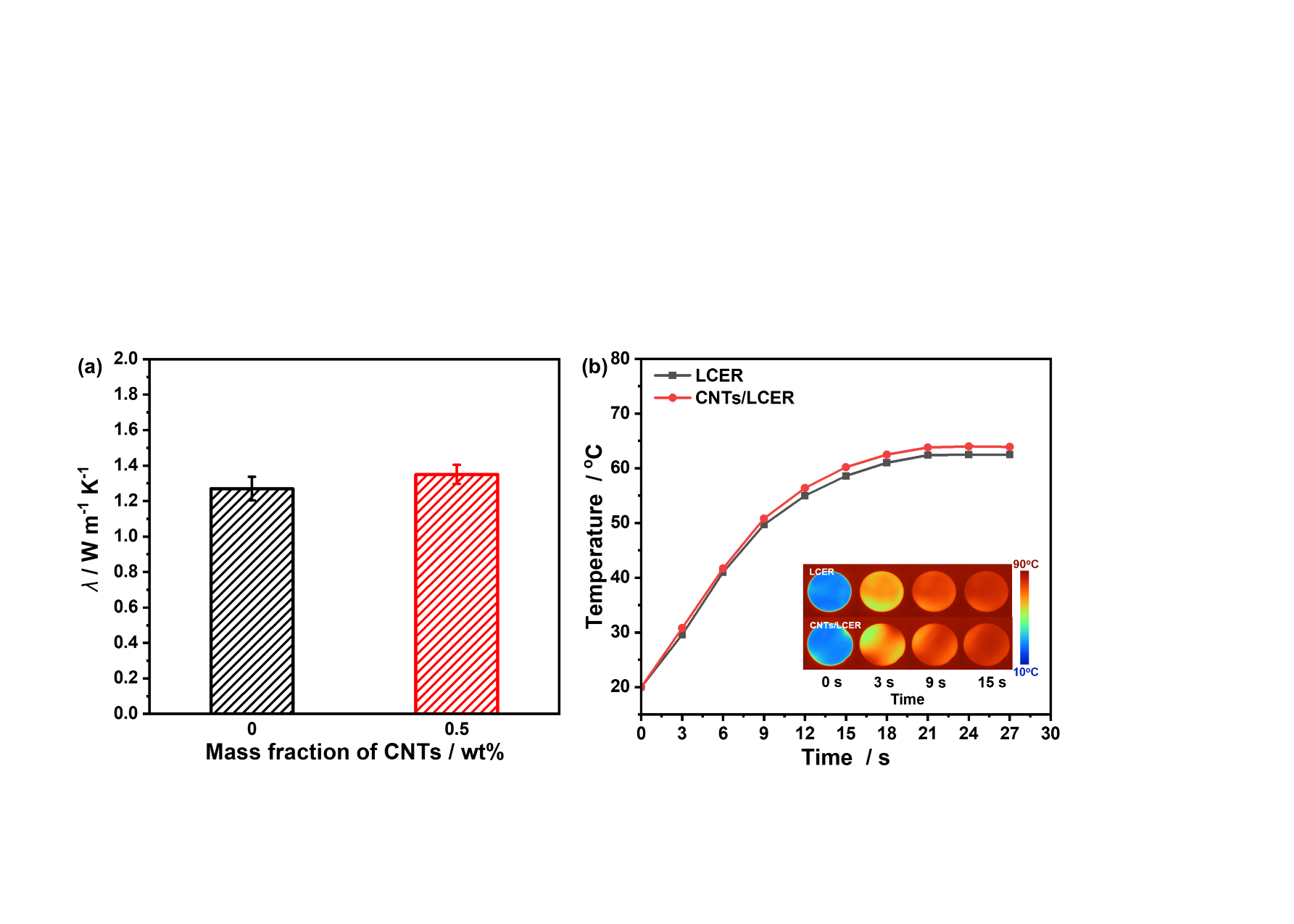
**

**Figure S5.** *λ* of LCER and CNTs/LCER vitrimers (a); time-dependent temperature changes of LCER and CNTs/LCER vitrimers on a 100^o^C hot plate, inset is infrared thermal images of CNTs/LCER vitrimer (b)


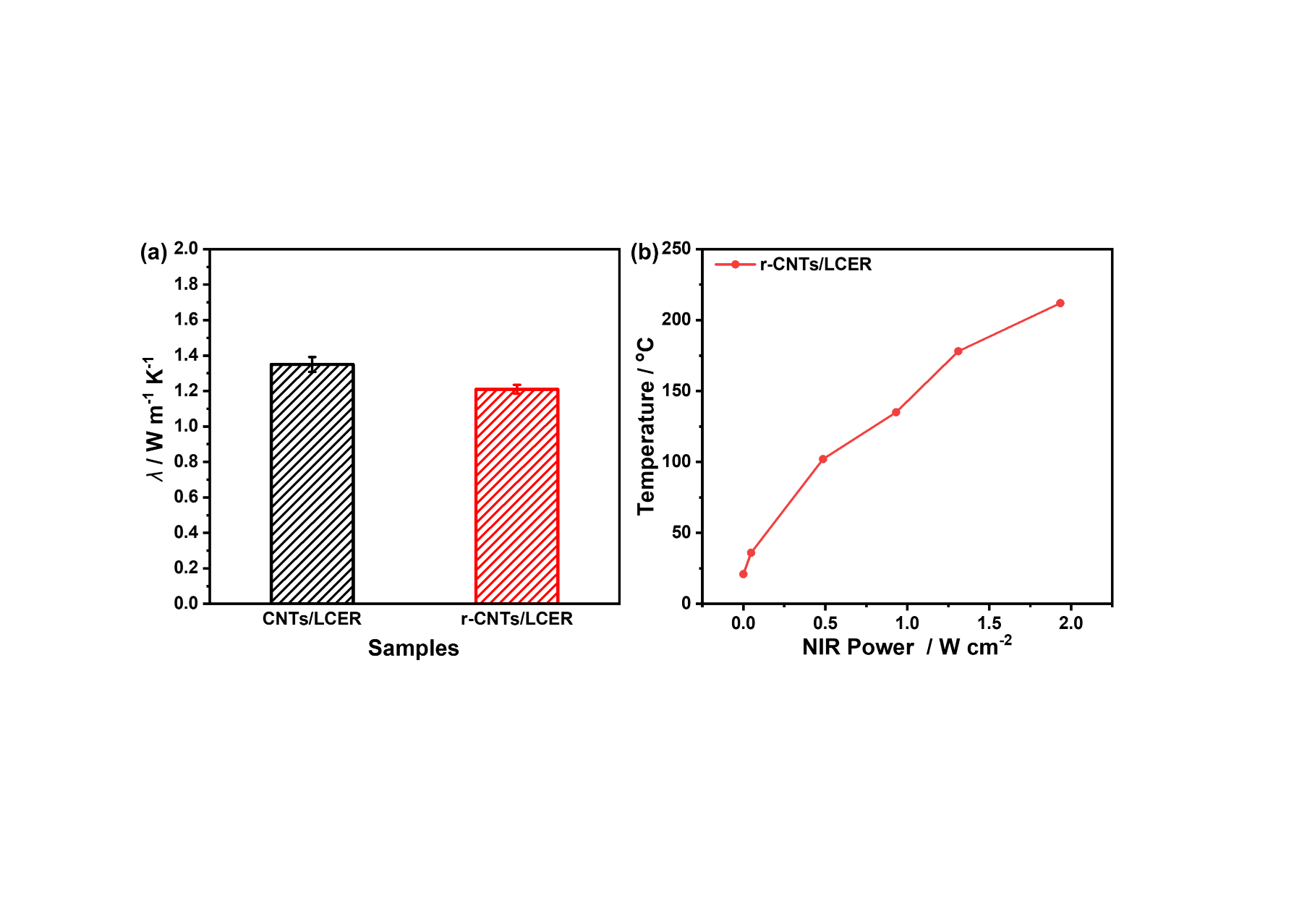


**Figure S6.** *λ* of original and recycled CNTs/LCER vitrimers (a); the surface temperatures of recycled CNTs/LCER vitrimer under different NIR (808 nm) densities of 0.05, 0.49, 0.93, 1.31, and 1.91 W cm^-2^ (b)
